# Distinct and Dissociable EEG Networks Are Associated With Recovery of Cognitive Function Following Anesthesia-Induced Unconsciousness

**DOI:** 10.3389/fnhum.2021.706693

**Published:** 2021-09-14

**Authors:** Alexander Rokos, Bratislav Mišić, Kathleen Berkun, Catherine Duclos, Vijay Tarnal, Ellen Janke, Paul Picton, Goodarz Golmirzaie, Mathias Basner, Michael S. Avidan, Max B. Kelz, George A. Mashour, Stefanie Blain-Moraes

**Affiliations:** ^1^Integrated Program in Neuroscience, McGill University, Montreal, QC, Canada; ^2^Neurology and Neurosurgery, McGill University, Montreal, QC, Canada; ^3^Cognitive Science, McGill University, Montreal, QC, Canada; ^4^School of Physical and Occupational Therapy, McGill University, Montreal, QC, Canada; ^5^Department of Anesthesiology, Center of Consciousness Science, University of Michigan Medical School, Ann Arbor, MI, United States; ^6^Department of Psychiatry, Perelman School of Medicine, University of Pennsylvania, Philadelphia, PA, United States; ^7^Department of Anesthesiology, Washington University School of Medicine, St. Louis, WA, United States; ^8^Deparment of Anesthesiology, Perelman School of Medicine, University of Pennsylvania, Philadelphia, PA, United States

**Keywords:** brain networks, functional connectivity, electroencephalography, cognitive function, anesthesia, partial least squares

## Abstract

The temporal trajectories and neural mechanisms of recovery of cognitive function after a major perturbation of consciousness is of both clinical and neuroscientific interest. The purpose of the present study was to investigate network-level changes in functional brain connectivity associated with the recovery and return of six cognitive functions after general anesthesia. High-density electroencephalograms (EEG) were recorded from healthy volunteers undergoing a clinically relevant anesthesia protocol (propofol induction and isoflurane maintenance), and age-matched healthy controls. A battery of cognitive tests (motor praxis, visual object learning test, fractal-2-back, abstract matching, psychomotor vigilance test, digital symbol substitution test) was administered at baseline, upon recovery of consciousness (ROC), and at half-hour intervals up to 3 h following ROC. EEG networks were derived using the strength of functional connectivity measured through the weighted phase lag index (wPLI). A partial least squares (PLS) analysis was conducted to assess changes in these networks: (1) between anesthesia and control groups; (2) during the 3-h recovery from anesthesia; and (3) for each cognitive test during recovery from anesthesia. Networks were maximally perturbed upon ROC but returned to baseline 30–60 min following ROC, despite deficits in cognitive performance that persisted up to 3 h following ROC. Additionally, during recovery from anesthesia, cognitive tests conducted at the same time-point activated distinct and dissociable functional connectivity networks across all frequency bands. The results highlight that the return of cognitive function after anesthetic-induced unconsciousness is task-specific, with unique behavioral and brain network trajectories of recovery.

## Introduction

Following the loss of consciousness from anesthesia, the brain can reconstitute its diverse range of cognitive functions, ranging from sensorimotor function to reasoning and logic, to memory. Although neural patterns associated with loss and recovery of consciousness have been characterized using functional brain dynamics and connectivity patterns (John et al., 2001; Lee et al., 2013; Blain-Moraes et al., 2015; Mashour and Hudetz, 2018), the neurocognitive trajectories associated with the reconstitution of cognition after anesthesia-induced unconsciousness are poorly understood. This can be attributed to the challenges associated with studying the neural correlates of the recovering brain after the clinical or experimental administration of general anesthesia. Previous studies have used cognitive tests administered pre– and post-anesthesia to compare recovery times from different anesthetics (Larsen et al., 2000), and to track the recovery times of various cognitive functions (N’Kaoua et al., 2002; Allampati et al., 2019). However, in these clinical studies with surgical patients, it is difficult to dissociate the effects of general anesthesia from the surgical intervention, which can adversely affect cognition through pain, inflammation, and analgesic confounds. In most experimental studies that isolate the effects of general anesthesia alone in healthy volunteers, the anesthetic protocols either just cross the threshold of unresponsiveness (Långsjö et al., 2005; Purdon et al., 2013; Blain-Moraes et al., 2015; Chennu et al., 2016; Scheinin et al., 2021), or induce a more profound unconsciousness for a short period of time (Banks et al., 2020). While these experimental anesthetic protocols may be suitable for modeling light sedation procedures (Allampati et al., 2019), they do not model general anesthesia for a major surgery, which animal studies have suggested can immediately and persistently impair cognition in the post-anesthetic period (Valentim et al., 2008; Carr et al., 2011; Avidan and Evers, 2016; Jiang et al., 2017). To study the neurocognitive recovery profiles under these circumstances, a protocol relevant to major surgery is required.

To address these limitations, we developed a protocol for healthy volunteers using a clinically relevant anesthetic regimen to induce unconsciousness without surgical intervention (Maier et al., 2017). High-density electroencephalography (EEG) was recorded during the administration of the anesthetic (15 min induction through propofol, 3 h maintenance with isoflurane), through the recovery of consciousness, and continued for 3 h after emergence. Isoflurane anesthesia is a halogenated ether and was selected over other anesthetics for its heterogenous molecular targets, which have a more profound effect on neural dynamics through multiple neurotransmitter receptor and channel systems. As a result, it has a slower offset in comparison to other anesthetics, theoretically providing the best opportunity to observe the differential recovery of cognitive function (Hemmings et al., 2019). Previous analysis of these data used source-localized spectral analysis, functional connectivity, and graph-theoretical approaches to characterize brain patterns during the recovery of consciousness and cognitive functions (Blain-Moraes et al., 2017). While global network efficiency distinguished between states of consciousness, it did not track the return of various cognitive functions. Conversely, alpha power in the superior parietal lobule only returned to baseline 90-min after recovery of consciousness, paralleling mean discharge-readiness times in the recovery room (Mashour et al., 2012). This source-localized spectral characteristic may be a biomarker for functional brain network recovery after anesthesia; however, the specific network mechanisms underlying the recovery of individual cognitive functions remain unknown. Two families of intrinsic coupling have been used to investigate the brain connectome using neurophysiological data: envelope coupling (also referred to as amplitude coupling) and phase coupling (Engel et al., 2013). Previous analyses of this dataset have shown that phase coupling, specifically, weighted phase lag index (wPLI) for the assessment of brain functional connectivity (Vinck et al., 2011), can characterize anesthetic-related changes in brain network function (Blain-Moraes et al., 2017; Duclos et al., 2021). We hypothesized that brain networks calculated with wPLI would similarly reveal anesthetic-related changes in cognitive function after recovery of consciousness.

Thus, the objective of the present study was to investigate network-level changes in functional brain connectivity associated with the recovery and return of six cognitive functions after anesthesia: attention, sensorimotor function, memory, reasoning and logic, abstract thinking, and maximal speed of cognitive processing. Since source localization or neuroimaging was not used in this study, the term “network” denotes a pattern of EEG- based functional connectivity. Multivariate partial least squares (PLS) analysis was applied to behavioral cognitive assessments and functional connectivity networks derived from EEG, testing the hypothesis that distinct networks could be associated with the return of specific cognitive functions following deep general anesthesia.

## Materials and Methods

This study was conducted at the University of Michigan Medical School and approved by the Institutional Review Board (HUM0071578); written consent was obtained from all participants.

### Study Population

Participants in this study were the subset of 20 healthy volunteers from the Reconstructing Consciousness and Cognition (ReCCognition) study (NCT01911195) evaluated at the University of Michigan who were selected because they underwent EEG recording with a 128-channel montage. The full protocol for this investigation has been published (Maier et al., 2017). All participants were class 1 physical status according to the American Society of Anesthesiologists, between 20 and 40 years of age, and had a body mass index of <30. Participants were excluded if they were pregnant, had a history of obstructive sleep apnea, reactive airway, gastroesophageal reflux, asthma, epilepsy, neuropsychiatric disorders, history or current use of psychotropic medications, cardiac conduction abnormalities, history of adverse reactions to anesthesia as well as family history of neurologic, psychiatric, or adverse reactions to anesthesia. Pregnancy and illicit drug use were ruled out with both urine and blood analyses.

### Experimental Protocol

Participants were randomized to one of two groups: general anesthesia with propofol and isoflurane, or wakefulness.

#### Anesthesia Group

**Figure 1:**
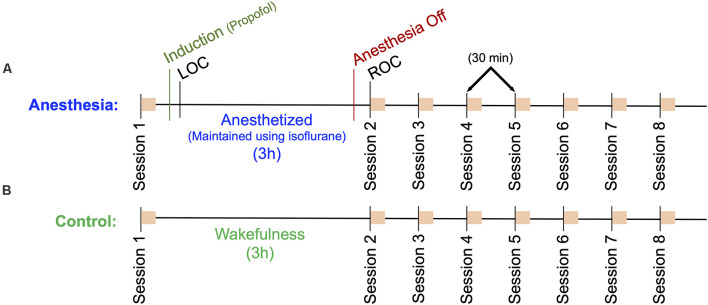
Outline of the experimental protocol. Eight sessions consisting of a battery of six neurocognitive tests are indicated as orange squares. **(A)** Anesthesia group protocol. A baseline cognitive test battery (Session 1) was followed by a 15-min propofol induction and a 3 h anesthesia using isoflurane. Session 2 cognitive tests commenced immediately upon recovery of consciousness (ROC), sessions 3–8 followed at 30-min intervals. **(B)** Control group protocol. A baseline cognitive test battery (Session 1) followed by a 3-h period of wakefulness, where participants read a book, watched a movie, or engaged in other wakeful behavior. Sessions 2–8 were then completed at 30 min intervals. High density EEG was recorded throughout the protocol for both groups.

Participants in the anesthesia group (*n* = 10) were brought into the Operating Room, where they were outfitted with a 128-channel EEG system (Electrical Geodesics, Inc., Eugene, OR, USA), and standard anesthesia monitors (electrocardiogram, non-invasive blood pressure cuff, pulse oximeter). In a seated position, participants completed a computerized neurocognitive test battery comprised of six independent cognitive tests (session 1). Upon completion, participants were moved to a supine position, and anesthesia was induced with a stepwise increase in propofol: 100 μg/kg/min for 5 min; 200 μg/kg/min for 5 min; 300 μg/kg/min for 5 min. During induction, participants followed a series of auditory commands delivered every 30 s to squeeze their right or left hand twice (right or left randomly delivered). Loss of consciousness (LOC) was defined as the first time the participant failed to respond to two consecutive commands. After induction, unconsciousness was maintained by inhaling 1.3 age-adjusted minimum alveolar concentration (MAC)—the concentration required to prevent movement in response to surgical stimuli in 50% of the population—of isoflurane. After 3 h, isoflurane was discontinued, and participants again listened to the same series of auditory commands every 30 s. Recovery of consciousness (ROC) was defined as the first time the participant responded to two consecutive auditory commands. At ROC, defined as *t* = 0, participants were returned to a seated position and repeated the computerized neurocognitive test battery (session 2). Neurocognitive testing was repeated at *t* = 30, 60, 90, 120, 150 and 180 min (sessions 3–8; Figure 1A).

#### Control Group

Participants in the control group (*n* = 10) were brought into a quiet room, where they were outfitted with the same EEG system as the Anesthesia group, *n* = 1 with 64 channels and *n* = 9 with 128 channels. This group followed the same experimental protocol, however, instead of being anesthetized, these participants remained awake by reading or watching television. They were instructed to avoid napping and were monitored by a research assistant to ensure compliance (Figure 1B).

### Neurocognitive Testing

During each of the eight neurocognitive testing sessions, participants completed a series of six computerized tests selected from the Cognition test battery (Basner et al., 2015) that reflect a broad range of cognitive domains, ranging from sensory-motor speed to complex executive functions. The test order was randomized across subjects, but each subject received the tests in the same order across sessions. We briefly describe the six tests chosen for this study in Table 1; for full details, see Maier et al. (2017).

**Table 1:** Neurocognitive tests within one testing session (Basner et al., 2015).

Cognitive function	Test	Description	Mean duration (seconds)
Sensorimotor speed	Motor Praxis (MP)	Sensorimotor speed was measured by asking participants to click on 20 consecutive squares that appear randomly on the screen (Basner et al., 2015), each successive square was smaller and thus more difficult to track.	39.60 ± 29.25
Spatial learning and memory	Visual Object Learning Test (VOLT)	This task measured the participant’s memory for complex figures by asking them to memorize 10 three-dimensional figures. Participants were then asked to distinguish the 10 memorized objects from a larger set of 20.	112.91 ± 24.22
Working memory	Fractal-2-Back (F2B)	Participants looked at a sequential 750 ms display of 62 fractal objects and were asked to press the spacebar when the current stimulus matched that displayed two figures before.	126.15 ± 13.21
Abstraction, concept formation	Abstract Matching (AM)	This test required participants to discern general rules from discrete examples, measuring executive function dependent on abstraction and concept formation. Participants were shown two pairs of objects that varied by perceptual dimensions and were asked to classify a target object as belonging to one of the two pairs.	118.46 ± 53.63
Vigilant attention	Psychomotor Vigilance Test (PVT)	This 3-min version of the PVT records reaction times to visual stimuli that occur at random inter-stimulus intervals. Subjects were instructed to monitor a box on the screen, and hit the space bar as fast as possible once a millisecond counter appears in the box and starts incrementing.	201.37 ± 19.62
Visual search, spatial memory, paired associate learning, sensory-motor speed	Digital Symbol Substitution Test (DSST)	Participants were required to refer to a displayed legend to decode specific symbols with each of the numbers from 1 to 9; during testing, a symbol appeared on the screen, and participants were asked to select the corresponding number as quickly as possible. The test duration was fixed at 90 s, and the legend key was randomly reassigned with each administration.	110.85 ± 29.24

### Electroencephalography Data Acquisition and Preprocessing

After fitting each participant with a 64- or 128-channel electrode net, impedances were brought to below 50 kΩ as per manufacturer recommendations. Data were sampled at 500 Hz, and all channels were referenced to the vertex. Throughout each recording, an investigator experienced in reading EEG visually monitored the data to ensure signal integrity. EEG data was bandpass filtered between 0.1 and 50 Hz and re-referenced to an average reference. Epochs and channels identified as containing non-physiological artifacts were removed. Independent component analysis (ICA) was used to remove electrooculogram (EOG) artifacts. Data were segmented by each of the six cognitive tests, within each of the eight recording sessions, for a total of 48 epochs per participant. Participants with too much electrophysiological noise or an incomplete set of neurocognitive tests were eliminated from the participant pool, yielding a final analysis sample of *n* = 8 for the Anesthesia group and *n* = 6 for the Control group. To facilitate the comparison of EEG networks across participants, the set of scalp electrodes common to all participants after data cleaning were identified, yielding a final set of 94 EEG channels that were used in the subsequent analyses.

### Electroencephalographic Network Analysis

We divided the EEG signal into five frequency bands—delta (1–4 Hz), theta (4–8 Hz), alpha (8–13 Hz), beta (13–30 Hz), and gamma (30–50 Hz)—using Butterworth band-pass filtering. We then constructed a functional brain network for each frequency band within each analysis epoch using the weighted phase lag index (WPLI; Vinck et al., 2011), which reduces the effects of EEG volume conduction.


(1) $$wPLI_{ij}=\frac{\left|E\left\{J(C_{ij})\right\}\right|}{E\left\{\left|J(C_{ij})\right|\right\}}=\frac{\left|E\left\{\left|J(C_{ij})\right|sgn(J(C_{ij}))\right\}\right|}{E\left\{\left|J(C_{ij})\right|\right\}}$$

where *J(C_ij_)* is the imaginary part of cross-spectrum *C_ij_* between signals *i* and *j*. The cross-spectrum *C_ij_* is defined as Z_i_Z^*^_j_, where *Z_i_* is the complex value Fourier spectra of signal *i* and Z^*^_j_ is the complex conjugate of *Z_j_*. WPLI ranges between 0 and 1: when the phase of one signal always leads or lags that of the other (i.e., if Pr{*sgn(J(C_ij_))* = 1 *or* −1}), then *WPLI_ij_* equals 1; when the phase lead and lag relationship between the two signals is random, then *WPLI_ij_* equals 0.

### Partial Least Squares (PLS) Analysis

Changes in functional connectivity patterns across the groups and conditions were assessed using mean-centering Partial Least Squares (PLS) analysis (McIntosh and Mišić, 2013). This multivariate technique detects the combination of groups/conditions and spatiotemporal patterns of neural activity that optimally relate to each other. This analysis enabled the isolation of networks of functional connectivity that collectively covaried with experimental manipulations. It also enabled the identification of the dominant, data-driven patterns without needing to specify *a priori* hypotheses about the differentiation between groups and conditions, or the specific spatiotemporal profiles of these differences.

PLS can be used to relate two “blocks” or sets of variables to each other (McIntosh and Lobaugh, 2004). We conducted three variations of the PLS analysis, contrasting: (1) WPLI matrices of the anesthesia and control groups during cognitive tests; (2) WPLI matrices of all testing sessions and all cognitive tests within only the anesthesia group; and (3) WPLI matrices from different sessions within a single cognitive task, within only the anesthesia group. The first variation assessed if changes in functional connectivity networks over time were significantly different between control and anesthesia groups. This analysis included WPLI matrices from all participants (anesthesia and control), all frequency bandwidths, and all analysis epochs (eight sessions, six cognitive tests). The second variation identified networks of functional connectivity that related to the recovery of cognitive function after anesthetic-induced unconsciousness. This analysis included WPLI matrices from only the anesthesia group, all five frequency bandwidths, and all analysis epochs (eight sessions, six cognitive tests). The third variation identified networks that differentiated the time-varying recovery of each of the six cognitive tests. Separate PLS analyses were conducted for each of the six cognitive tests. Each variation three analysis included WPLI matrices from the anesthesia group only, a single frequency band, all sessions, and a single cognitive test.

For each variation of the PLS analysis, the covariance matrix between the two sets of variables was computed, and decomposed into mutually orthogonal “latent variables” using singular value decomposition (SVD; Eckart and Young, 1936). Each latent variable was expressed as a vector of design weightings, a vector of functional connectivity weightings, and a scalar singular value (*s*). The two vectors reflect a symmetrical relationship between the experimental design component most related to the differing functional connectivity values on the one hand, and the optimal (in the least squares sense) network of connectivity related to the identified experimental design components on the other. In other words, the elements of the design weighting vectors represent a contrast of maximal covariance between groups and/or conditions, while the functional connectivity weightings represent a pattern of WPLI connections and frequencies that maximally expressed that contrast. The singular value reflects the covariance between the design variables (groups and conditions) and functional connectivity variables (WPLI) that are captured by each latent variable. The effect size ranges from 0 to 100% and reflects the proportion of cross block covariance accounted for by each latent variable. It is calculated as the ratio of the square of its singular value to the sum of all squared singular values derived from the decomposition (Berman et al., 2014; Mišić et al., 2016). A design salience (optimal contrast) is depicted for each latent variable. This variable ranges from −1 to +1 and represents the overall multivariate pattern of covariance attributable to each condition (design weighting). The design saliences across all conditions of any given contrast sum to zero.

The statistical significance of each latent variable (i.e., each design salience contrast) was determined using permutation tests. The group and condition labels of the WPLI matrices were randomly permuted and the new data were subjected to SVD as described above, yielding a new set of singular values. These singular values were associated with the null hypothesis that there is no association between the EEG functional connectivity matrix and the group or condition. This procedure was repeated 500 times to generate a sampling distribution of singular values under the null hypothesis. The *p*-value for each latent variable was estimated as the probability that singular values from the distribution of permuted samples exceeded that from the original non-permuted data.

The reliability of the spatiotemporal patterns associated with each effect was estimated by calculating the standard errors of each functional connectivity weighting using bootstrap resampling (Efron and Tibshirani, 1986). Bootstrap samples were generated by randomly sampling participants with replacement, while their group and condition assignments were preserved, and subjecting the new data to SVD as described above. This process was repeated 500 times, generating a bootstrap distribution for each functional connectivity weighting. The goal of this process was to identify weightings that were stable regardless of which participants were included in the sample. Bars that possessed 95% confidence intervals that did not contain 0 were considered driving factors in the latent variable (i.e., significant and reliable contributors to the design salience) and have been marked with * in the figures.

The magnitude and stability of the connectivity of each pair of electrodes to the overall network were assessed by calculating the bootstrap ratio (*b*_i_), defined as the functional connectivity weighting divided by its bootstrap-estimated error. If we assume that the bootstrap distribution is approximately unit normal (Efron and Tibshirani, 1986), the bootstrap ratios are approximately equivalent to a *Z*-score. Bootstrap ratios were thresholded at the 99% confidence interval to generate a network of functional connectivities between EEG electrodes that reliably express the statistical effect (pattern of functional connectivity variance) captured by the latent variable. Positive bootstrap ratios indicate that the associated functional connectivity network expresses the contrast in the depicted orientation whereas negative bootstrap ratios indicate that the network expresses the contrast in the opposite orientation (Mišić et al., 2016).

### Statistical Analysis

In order to compare cognitive recovery trajectories across participants, scores for all cognitive tests were normalized to 0 (performance at baseline). Normalized scores for each cognitive test were averaged for each recording session across anesthesia and control groups. To determine if there was a significant change in performance across the experimental sessions, a repeated-measures analysis of variance (ANOVA) with a Greenhouse-Geisser correction was performed for each cognitive test, with differences considered significant at *p* < 0.05. To assess when performance on each cognitive test returned to baseline, *post hoc* pairwise *t*-tests tests using the Bonferroni correction were used to identify the sessions with significantly different cognitive scores; recovery of performance was marked at the first session where scores were not significantly different from baseline.

## Results

### Temporal Recovery of Cognitive Performance Varies by Task After Anesthesia-Induced Unconsciousness

**Figure 2:**
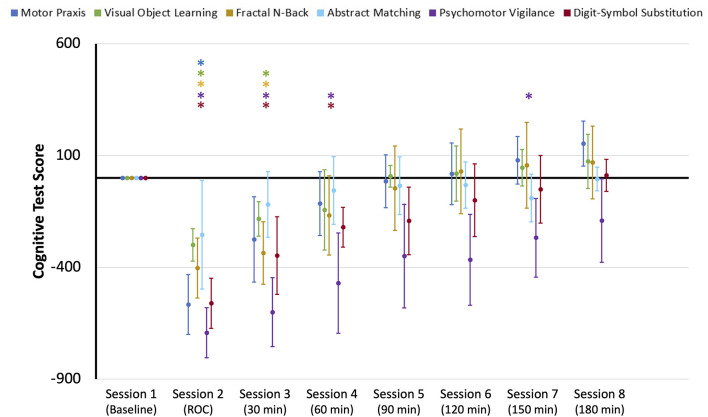
Cognitive test performance scores for the eight sessions of the neurocognitive test battery: baseline, upon recovery of consciousness (ROC), and at six 30-min intervals for 3 h post-ROC. Scores were normalized to baseline performance (0). Error bars indicate standard deviation, and * indicates sessions where scores for each cognitive test were significantly different from baseline.

Upon recovery from anesthesia-induced unconsciousness, performance scores significantly decreased from baseline for all cognitive tests, with the exception of Abstract Matching. Performance on the remaining five cognitive tests returned to baseline at varying rates (Figure 2). Motor Praxis scores returned within 30 min; Visual Object Learning and Fractal-n-Back scores returned within 60 min; Psychomotor Vigilance and Digital-Symbol Substitution Task scores returned within 90 min. In the control group, a significant difference in performance scores was only found for the VOLT test at session eight compared to session one (Supplementary Figure 1), demonstrating that the changes observed in the experimental group were a direct result of anesthesia-induced unconsciousness, and not related to fatigue or learning effects. These results parallel the cognitive recovery trajectories of participants in the full ReCCognition study, wherein 30 individuals showed differential recovery times following anesthesia of the speed and accuracy of tasks associated with attention, complex scanning and visual tracking, working memory, and executive function (Mashour et al., 2020).

### Networks Associated With Cognition Are Significantly Altered After Anesthesia

The first variation of the PLS analysis assessed if changes in cognitive networks over time were significantly different between control and anesthesia groups. Three latent variables (LV) emerged as significant (LV1: *p* < 0.0005, effect size (ES) = 87%, LV2: *p* = 0.04, *ES* = 3%, LV3: *p* = 0.03, *ES* = 0.003%). As latent variables 2 and 3 only accounted for 3% and 0.003% of the cross block covariance (as determined by effect size calculations) respectively, they were excluded from subsequent analysis.

**Figure 3:**
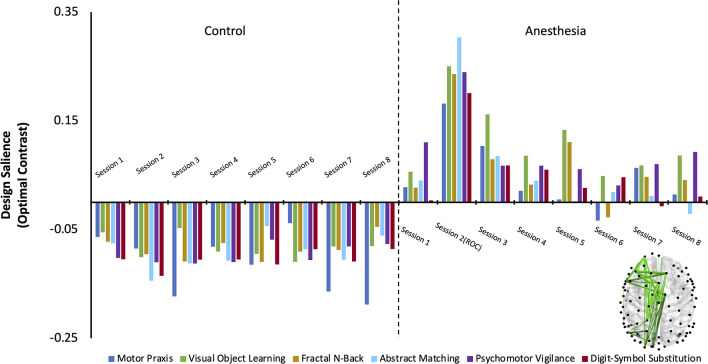
The dominant latent variable (LV = 1) capturing the network of maximal functional connectivity covariance between groups (Control and Anesthesia) across all eight testing sessions and all six cognitive tasks (PLS analysis, variation 1). Permutation testing confirmed the statistical significance of the entire design salience (*p* < 0.0005), and the attributable proportion of cross block covariance (ES) was calculated to be 87%. Connections that reliably express the contrast were identified using bootstrap ratios thresholded at 99% (see “Materials and Methods” section) and are mapped on the brain.

Latent variable 1 was associated with a network that significantly differentiated anesthesia and control groups (Figure 3). Functional connectivity networks in the control group had negatively loaded design weightings, while those in the anesthesia group had predominantly positively loaded design weightings. This contrast captures a functional connectivity network that maximally varied between the control and anesthesia groups across all testing sessions and demonstrates that networks activated during each cognitive task are significantly altered after a period of profound anesthesia-induced unconsciousness. The anesthesia group also expressed a significant session × task interaction, which was not observed in the control group. This interaction was driven by the large contrast in networks across all tasks upon recovery of consciousness, indicating that cognitive networks are maximally altered at this point in the recovery trajectory.

### Networks Activated by Cognitive Tasks Return to Their Baseline State 30–60 min Following Recovery of Consciousness

One latent variable emerged as significant (LV1: *p* = < 0.0005; *ES* = 74%) in the second variation of PLS analysis (i.e., anesthesia only), which identified the functional connectivity network that maximally varied across the recovery of cognitive function after anesthetic-induced unconsciousness. The design salience associated with this latent variable is positively loaded and significantly different from baseline across all cognitive tasks immediately upon recovery of consciousness (session 2) indicating that the multivariate pattern of covariance is maximally attributable to this testing session (i.e., the maximal variance in the WPLI of the associated network is seen immediately upon recovery of consciousness). Bootstrap ratios for the associated network are positive, indicating that the functional connectivity within the associated network is stronger at this stage of recovery relative to baseline (the network expresses the contrast in the depicted orientation). The non-zero confidence intervals associated with each cognitive task at this testing session indicate that this condition (session 2) most reliably drives/expresses the multivariate contrast seen within participants exposed to anesthesia.

**Figure 4:**
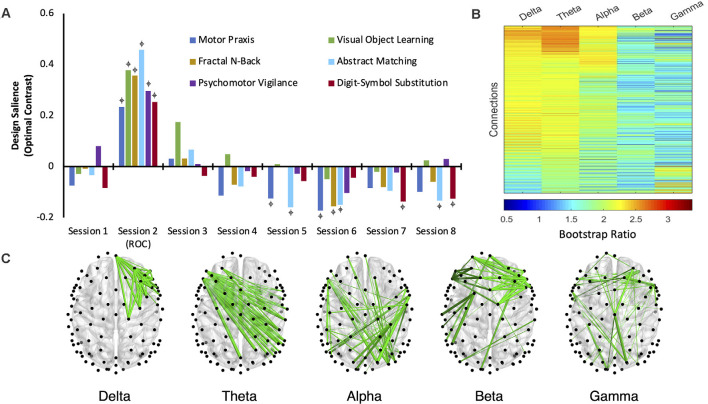
The dominant latent variable (LV = 1) capturing the network of maximal functional connectivity covariance across the anesthetic protocol (five frequency bands, eight cognitive testing sessions, consisting of six cognitive tasks; PLS analysis, variation 2). **(A)** Permutation testing identified a significant design salience (optimal contrast) that captured a session × task interaction depicting the maximal covariance of function related to the recovery of cognitive function after anesthetic-induced unconsciousness (*p* < 0.0005) with an attributable proportion of cross block covariance (ES) of 74%. 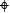 = non-zero bootstrap-estimated 95% confidence interval. **(B)** Bootstrap ratios (BSRs) for each connection (pair of electrodes) associated with each frequency band. The higher the BSR magnitude, the more reliably the connection expressed the contrast in its present orientation (see “Materials and Methods” section). **(C)** The top 1% of connections that reliably express the contrast, determined by thresholding the BSRs.

Brain networks associated with each cognitive task are not differentiable from the baseline state at sessions 3 and 4 (Figure 4A). Thus, networks associated with all six cognitive tasks returned to their baseline pattern between 30 min and 1 h following recovery of consciousness. While the networks associated with some cognitive tests expressed a significant, negatively loaded design salience between sessions 5–8, no changes were consistent over consecutive sessions.

Across all five frequency bandwidths, changes in cognitive networks post-ROC were most robustly expressed in delta, theta, and alpha, with functional connectivity in theta having the highest bootstrap ratios (Figure 4B). This indicates that networks isolated from the theta band (Figure 4C) most reliably express the contrast captured in this latent variable across sessions following exposure to anesthesia. The salience network associated with theta demonstrates that the disruption of inter-hemispheric, long-range connectivity is associated with the significant changes in cognitive function immediately upon recovery of consciousness (session 2).

### Recovery of Cognitive Functions Activates Distinct and Dissociable Networks

**Figure 5:**
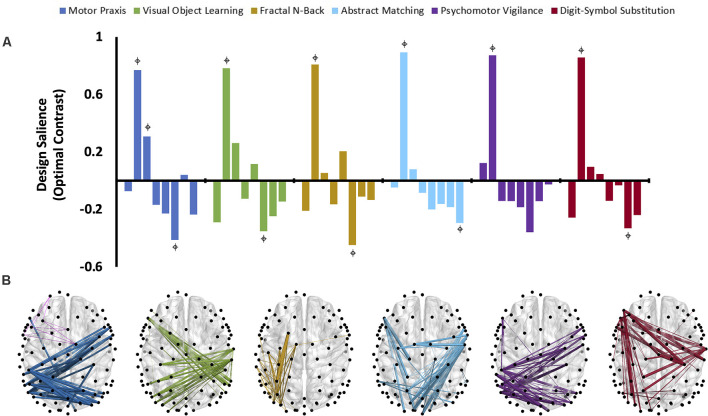
The dominant latent variables (LV = 1 in all cases) capturing the distinct networks of maximal functional connectivity covariance across all eight cognitive testing sessions corresponding to each cognitive task in the alpha bandwidth (PLS analysis, variation 3). **(A)** Design saliences associated with network changes for each cognitive test across all eight sessions, where each bar corresponds to a single cognitive testing session. 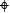 = non-zero bootstrap-estimated 95% confidence interval. Permutation-estimated *p*-values and proportions of attributable cross block covariance (ES) associated with the significant latent variable are included in Table 2. **(B)** The top 1% of connections corresponding to each cognitive test that reliably express the contrast, determined by thresholding the bootstrap ratios.

**Figure 6:**
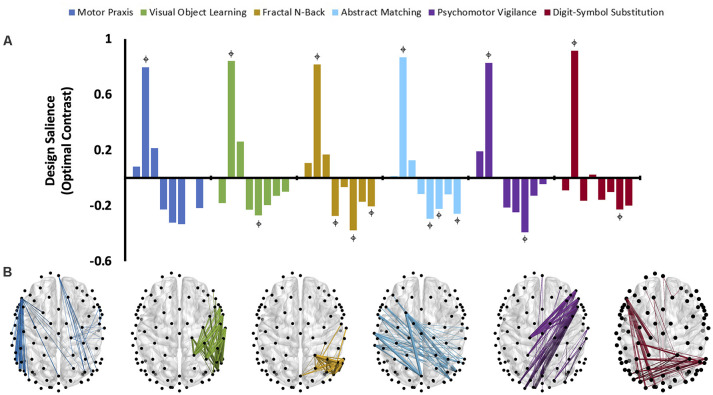
The dominant latent variables (LV = 1 in all cases) capturing the distinct networks of maximal functional connectivity covariance across all eight cognitive testing sessions corresponding to each cognitive task in the theta bandwidth (PLS analysis, variation 3). **(A)** Design saliences associated with network changes for each cognitive test across all eight sessions, where each bar corresponds to a single cognitive testing session. 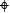 = non-zero bootstrap-estimated 95% confidence interval. Permutation-estimated *p*-values and proportions of attributable cross block covariance (ES) associated with the significant latent variable are included in Table 2. **(B)** The top 1% of connections corresponding to each cognitive test that reliably express the contrast, determined by thresholding the bootstrap ratios.

The third variation of PLS analysis identified functional connectivity networks that maximally varied across testing sessions (i.e., across recovery from anesthesia). These contrasts differentiated the time-varying recovery of each cognitive test. Within alpha and theta bandwidths, one latent variable was significant for each cognitive test, indicating that significant changes occurred in patterns of networks associated with each cognitive test over the eight testing sessions (Figure 5A, Figure 6A). The effect size for all cognitive tests is high (Table 2), indicating that a large proportion of the cross block covariance is captured by the latent variable. The Motor Praxis Task has a relatively lower effect size (alpha *ES* = 61%, theta *ES* = 68%) compared to the other five tasks. In this task, other patterns of network changes account for a large degree of the variability expressed by the network across time in addition to the pattern captured by the significant latent variable.

**Table 2:** Partial Least Squares (PLS) variation three permutation and bootstrapping results for alpha and theta bands.

Cognitive test	Latent variable 1 *p* - value (design salience statistical significance) and effect size (proportion of cross block covariance captured)
Motor Praxis (MP)	Alpha: *p* = 0.006, *ES* = 61% Theta: *p* = 0.008, *ES* = 68%
Visual Object Learning Test (VOLT)	Alpha: *p* = 0.008, *ES* = 80% Theta: *p* = 0.002, *ES* = 90%
Fractal-2-Back (F2B)	Alpha: *p* = 0.002, *ES* = 84% Theta: *p* < 0.0005, *ES* = 93%
Abstract Matching (AM)	Alpha: *p* < 0.0005, *ES* = 91% Theta: *p* < 0.0005, *ES* = 95%
Psychomotor Vigilance Test (PVT)	Alpha: *p* = 0.008, *ES* = 87% Theta: *p* = 0.002, *ES* = 92%
Digital Symbol Substitution Test (DSST)	Alpha: *p* = 0.002, *ES* = 81% Theta: *p* = 0.008, *ES* = 91%

Design saliences and thresholded associated functional connectivity networks corresponding to each cognitive test in the alpha bandwidth are presented in Figure 5, and those for the theta bandwidth in Figure 6. Data associated with the latent variables for delta, beta, and gamma bandwidth are included in Supplementary Figures 2–4. Across all analyses, the largest contrast in design salience occurs at session two, immediately upon recovery from anesthesia-induced unconsciousness. Bootstrapping provided 95% confidence intervals that were consistently non-zero at session two across all cognitive tasks and frequency bands. As expected, this indicates that each of these distinct cognitive networks is maximally altered upon recovery of consciousness and that this session is reliably, and significantly, contributing to the multivariate pattern of covariance. The congruent directionality of the design salience weightings (positively loaded) and bootstrap ratios (positive values) upon recovery of consciousness indicates that functional connectivity within these distinct networks is significantly stronger than at baseline. This may suggest that these networks play a significant role in the recovery of the specific cognitive function associated with the cognitive test driving the contrast. Strikingly, the alpha and theta networks associated with cognitive recovery are distinct across each of the six cognitive tests (Figure 5B, Figure 6B). In the alpha bandwidth, all tasks except the Psychomotor Vigilance Test have a significant negative contrast during one session post-ROC (Figure 5A). In the theta bandwidth, all tasks except the Motor Praxis Task have one to three sessions that express significant negative contrast post-ROC (Figure 6A). Given that the bootstrap ratios were positive values, the negatively loaded design salience bars can be interpreted as significantly weaker functional connectivity within the associated networks at this point in recovery relative to baseline and to the significantly positive design saliences seen at session 2.

## Discussion

In this study with healthy adults exposed to a clinically relevant anesthesia regimen, we demonstrated that six distinct cognitive functions follow different temporal trajectories of recovery following anesthesia, with certain cognitive functions returning to baseline only 90–180 min following the recovery of consciousness. These results were supported by a larger study of 60 individuals (including those in this study) showing that the rates of recovery statistically differed among cognitive domains upon recovery from anesthetic-induced unconsciousness (Mashour et al., 2020). Importantly, we found that brain networks associated with cognitive tasks are significantly altered after a period of profound anesthesia-induced unconsciousness, return to their baseline state 30–60 min following recovery of consciousness, and are distinct and dissociable across various cognitive functions.

Globally, the results from several variations of the PLS analysis reflect well-known effects of anesthesia, namely, the impairment and gradual recovery of cognitive functions following anesthetic-induced unconsciousness (Larsen et al., 2000; Chen et al., 2001). The results from our data-driven multivariate EEG analysis provide evidence for network-level disturbances that accompany the anesthetic-induced change in cognitive function. Like cognitive task performance, the brain networks are maximally altered immediately upon recovery of consciousness. Although network-level alterations return to baseline 30–60 min following ROC, cognitive test performance did not recover until 90–180 post-ROC. These findings are consistent with our previous analyses of this dataset, which have shown that after ROC, neurophysiological characteristics such as source-localized alpha power and graph theory characteristics of brain networks return to baseline prior to the full recovery of cognitive functions (Blain-Moraes et al., 2017).

Functional connectivity in theta bandwidth was most strongly associated with brain network contrasts in the 3 h after recovery of consciousness, with long-range interhemispheric connections expressing maximal contrast immediately upon recovery of consciousness. These findings add to the large body of literature demonstrating anesthetic suppression of long-range functional connectivity (Boveroux et al., 2010; Schröter et al., 2012; Barttfeld et al., 2015). Moreover, theta band activity in frontal electrodes has been positively linked to working memory demand (Sauseng et al., 2005; Grunwald et al., 2014), and theta oscillations are implicated in the coordination and integration of various cognitive processes during working memory intensive tasks (Sarnthein et al., 1998; Sauseng et al., 2010). We, therefore, suggest that the increased theta connectivity network observed during cognitive tests performance following recovery of consciousness may represent increased recruitment of cognitive resources to compensate for the major perturbation of anesthesia. Networks isolated from the alpha bandwidth also reliably express the contrast across sessions following exposure to anesthesia. As alpha band oscillations have been related to the inhibition of brain activities that are not involved in a cognitive task (Klimesch et al., 2007; Jensen and Mazaheri, 2010; Uusberg et al., 2013), it is possible that the alpha recovery network maximizes a limited cognitive reserve by suppressing brain activities that are not required for the specific cognitive task. These results may reflect the complementary and compensatory roles of theta and alpha recovery networks in response to anesthesia-induced perturbations in cognitive functioning.

PLS analysis of the functional connectivity networks associated with specific cognitive tests across recovery from anesthesia isolated distinct and dissociable networks for each cognitive activity across all frequency bands. For example, in the alpha bandwidth, the motor praxis and psychomotor vigilance tasks were similarly associated with inter-hemisphere parietal connections and long-range right frontal to left parietal connections, while the visual object learning task was associated with similar parietal, but left frontal to right parietal long–range connections. The fractal n-back task was associated with short-range connections in the left parietal, temporal and occipital regions. Long-range connections associated with the abstract matching task converge in parietal regions, while long-range connections associated with the digital symbol substitution task converged in the left frontal regions. Strikingly, these networks do not overlap with the canonical network associated with specific cognitive functions [e.g., the VOLT primarily activates the frontal and bilateral anterior medial temporal cortices and the hippocampus (Jackson and Schacter, 2004); PVT recruits the prefrontal cortex, motor cortex, inferior parietal cortex and visual cortex (Basner et al., 2015)]. This does not necessarily indicate that these canonical networks are inactive. Rather, as PLS analysis foregrounds the contrast in networks across conditions, this indicates that the canonical networks exhibit similar levels of connectivity across the 3 h following recovery of consciousness. In other words, the changes in cognitive task performance in the 3 h post-ROC are not associated with changes in the strength or number of functional connections of the networks typically associated with these cognitive activities. Therefore, the present results, which show that specific networks are differentially activated according to cognitive tasks after recovery from anesthetic-induced unconsciousness, must be interpreted carefully. One possibility is that these networks reflect an adaptive, compensatory network associated with cognitive reserve: non-traditional cognitive networks that are recruited to maintain cognitive function when traditional networks are disrupted (Barulli and Stern, 2013). Another possibility is that these networks are inappropriately recruited for the cognitive task as the brain recovers from the functional connectivity disruption of anesthesia (Barttfeld et al., 2015). Indeed, each network also exhibits a negative salience 60–90 min post recovery of consciousness, potentially indicating an inhibition of these networks once baseline functional connectivity patterns are restored. This timeline is congruent with our prior findings that source-localized alpha power returns to baseline levels within 90 min of recovery of consciousness (Blain-Moraes et al., 2017), and with results from a large randomized control trial assessing different methods of monitoring intraoperative awareness, where mean discharge-readiness times in the recovery room were approximately 95 min (Mashour et al., 2012). Finally, these network changes may reflect residual effects of the prolonged exposure to anesthesia. Loss and recovery of consciousness associated with general anesthesia have been posited to be governed by the principle of “neural inertia”, a neurobiological process that maintains aroused and anesthetized states, and creates resistance to behavioral state transitions (Friedman et al., 2010). It is likely that, upon recovery of consciousness, the brain’s neural inertia maintains many of the network properties that existed immediately prior to the moment of return of behavioral responsiveness. As a result of this neural inertia, the major difference in functional connectivity networks observed during the cognitive tests conducted immediately upon recovery of consciousness may also reflect the characteristics of the networks involved in the return of consciousness.

Our study design offers a unique opportunity to examine isolated brain networks associated with specific cognitive functions. In healthy human studies, the assessment of functional connectivity patterns associated with higher-order cognitive tasks, such as executive function, is typically confounded by concomitant cognitive functions, such as attention. In this study, cognitive task performance is differentially affected by anesthesia, particularly in the minutes immediately after ROC. Loosely, anesthesia performs a temporal separation of cognitive function performance much like gel electrophoresis separates molecules, enabling us to individually examine the component parts. The fact that these isolated cognitive functional connectivity networks do not map onto canonical networks may prompt a reconsideration of the relationship between functional connectivity and cognition. Indeed, anesthesia has been successfully used as a tool to re-examine the relationship of functional connectivity to brain-based phenomena. In a recent study, cholinergic stimulation of the prefrontal cortex induced wakefulness in a rat model, despite continuous exposure to general anesthesia (Pal et al., 2020). Surprisingly, functional connectivity remained suppressed during the induced wakeful period in the presence of anesthetics, which suggested that the level of consciousness can be dissociated from cortical connectivity. Similarly, the findings of this study suggest that functional connectivity patterns may be dissociable from cognitive tasks and could prompt a reevaluation of the role of these connectivity measures in cognition.

This study has several limitations. First, the number of participants in the study is relatively low (*n* = 14). The results of PLS analysis are sensitive to signal/noise ratio (Cramer, 1993), which is decreased in this sample size by the high inter-subject variability of brain network activity after recovery from anesthetic-induced unconsciousness. However, the latent variables for variations one and two of the analysis accounted for 87% and 74% of the network covariance respectively, indicating the robustness of these results despite the smaller sample size. In the third variation of the PLS analysis, which identified networks that differentiated the time-varying recovery of each cognitive test, networks associated with motor praxis (theta and alpha) and visual object learning (alpha) explained less than 70% of the covariance. While the results are statistically significant, the latent networks associated with these cognitive tests should be interpreted with caution. Second, we use a single measure of functional connectivity—wPLI—to assess the functional coupling of brain networks during cognitive tests. Although we chose this measure due to its robustness against volume conduction (Vinck et al., 2011), this may bias our results, as other types of phase-based connectivity or families of connectivity (e.g., envelope-based connectivity) may identify different coupling patterns across cognitive tasks. For example, several recent studies have demonstrated that envelope- and phase-based measures of functional connectivity capture different and complementary relationships between brain regions, especially in the networks related to loss and recovery of consciousness (Siems and Siegel, 2020; Duclos et al., 2021). wPLI also only provides information about the strength, not the direction, of functional coupling, which may be investigated with other connectivity metrics such as directed phase lag index (Stam and van Straaten, 2012). Third, our functional connectivity networks were constructed based on sensor-level EEG data, with the nodes of the networks constrained to the fixed electrode positions of the EEG net. The data were not source localized, limiting the conclusions that can be drawn about the specific brain regions implicated in the networks associated with the recovery of cognitive function presented in our results. Finally, across all PLS analysis variations, the largest contrast was uniformly driven by the cognitive testing period immediately succeeding recovery of consciousness. While these results align with our expectation of maximal impairment upon return of responsiveness, the magnitude of this contrast dwarfs the subsequent sessions, potentially masking more subtle network changes that accompany the return to baseline cognitive performance up to 3 h after recovery of consciousness.

The present study investigated EEG network-level changes in functional connectivity associated with the recovery of six cognitive functions following a clinically relevant anesthesia regimen. Brain networks associated with cognitive tests were significantly altered in patients recovering from anesthesia compared to control subjects. Across 3 h post-ROC, the cognitive networks of participants recovering from anesthesia were most significantly altered in the theta bandwidth, particularly in long-range interhemispheric connections. Finally, different cognitive functions were associated with distinct and dissociable brain network changes across the 3-h recovery period. Collectively, these results demonstrate that cognitive functions have distinct temporal and network-level patterns of reconstitution following an anesthetic-induced loss of consciousness. Future studies should aim to validate these findings with neuroimaging techniques with an appropriate spatial resolution to identify the specific brain regions involved in the reconstitution of each cognitive activity following anesthesia.

## Data Availability Statement

The raw data supporting the conclusions of this article will be made available by the authors, without undue reservation.

## Ethics Statement

The studies involving human participants were reviewed and approved by University of Michigan Medical School. The patients/participants provided their written informed consent to participate in this study.

## Author Contributions

MA, MK, and GM conceived and designed the study. MB designed the cognitive tests and extracted cognitive test data. SB-M and GG acquired EEG data. VT, EJ, PP, GG, and GM served as the clinical anesthesiologists. AR and KB analyzed the data. AR, BM, CD, SB-M, and GM interpreted the data. AR, CD, and SB-M wrote the manuscript. All authors contributed to critical review of the manuscript. All authors contributed to the article and approved the submitted version.

## Conflict of Interest

The authors declare that the research was conducted in the absence of any commercial or financial relationships that could be construed as a potential conflict of interest.

## Publisher’s Note

All claims expressed in this article are solely those of the authors and do not necessarily represent those of their affiliated organizations, or those of the publisher, the editors and the reviewers. Any product that may be evaluated in this article, or claim that may be made by its manufacturer, is not guaranteed or endorsed by the publisher.
