# Supplementary material for: Distinct and Dissociable EEG Networks Are Associated With Recovery of Cognitive Function Following Anesthesia-Induced Unconsciousness
Source: Front Hum Neurosci. 2021 Sep 14;15:706693. doi: 10.3389/fnhum.2021.706693 (PMC8477048; doi:10.3389/fnhum.2021.706693)
Supplement: Supplementary file 1 [file Data_Sheet_1.docx]

Supplementary Material

# Supplementary Figures and Tables

## Supplementary Figures


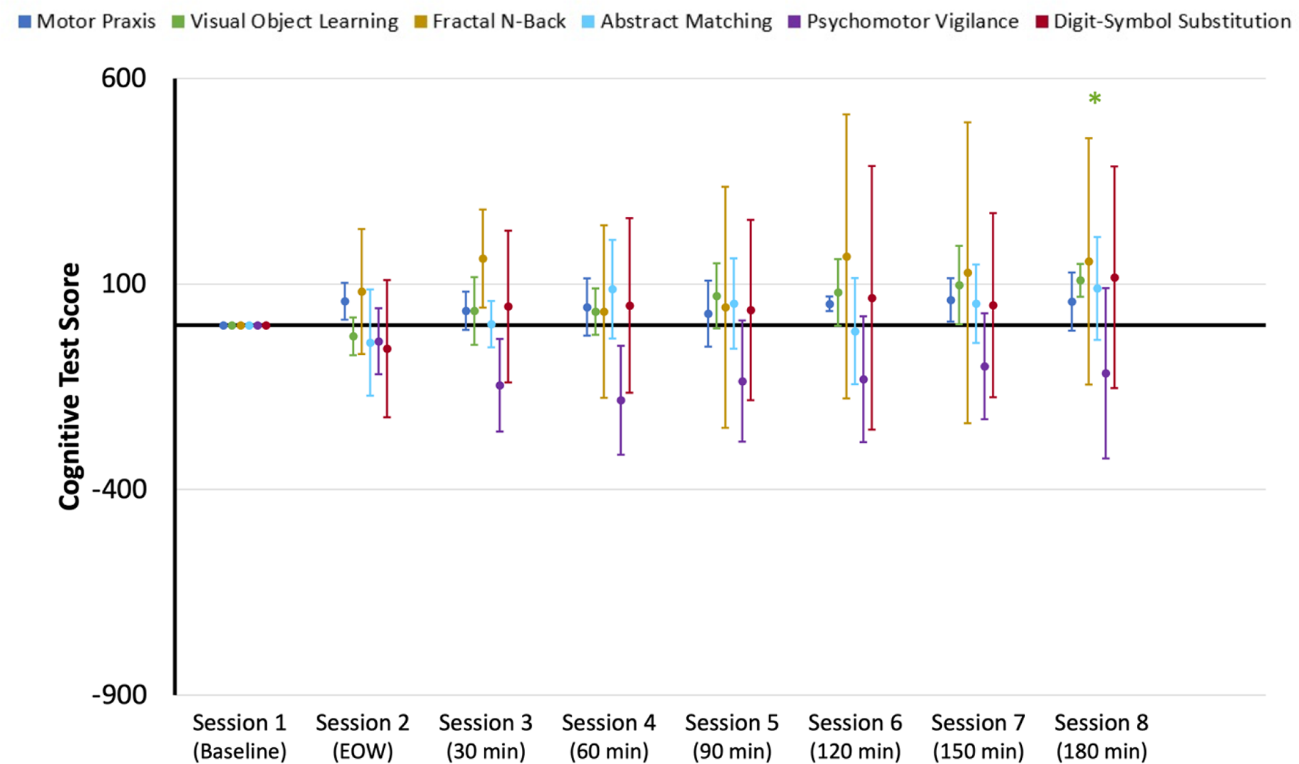


**Supplementary Figure 1.** Control group cognitive test performance scores. Mean scores and standard deviations are plotted for each of the eight sessions of the neurocognitive test battery: baseline (Session 1), end of wakefulness period (EOW, Session 2), and at six 30-minute intervals for three hours post-EOW (Sessions 3-8). Scores were normalized to baseline performance (0).


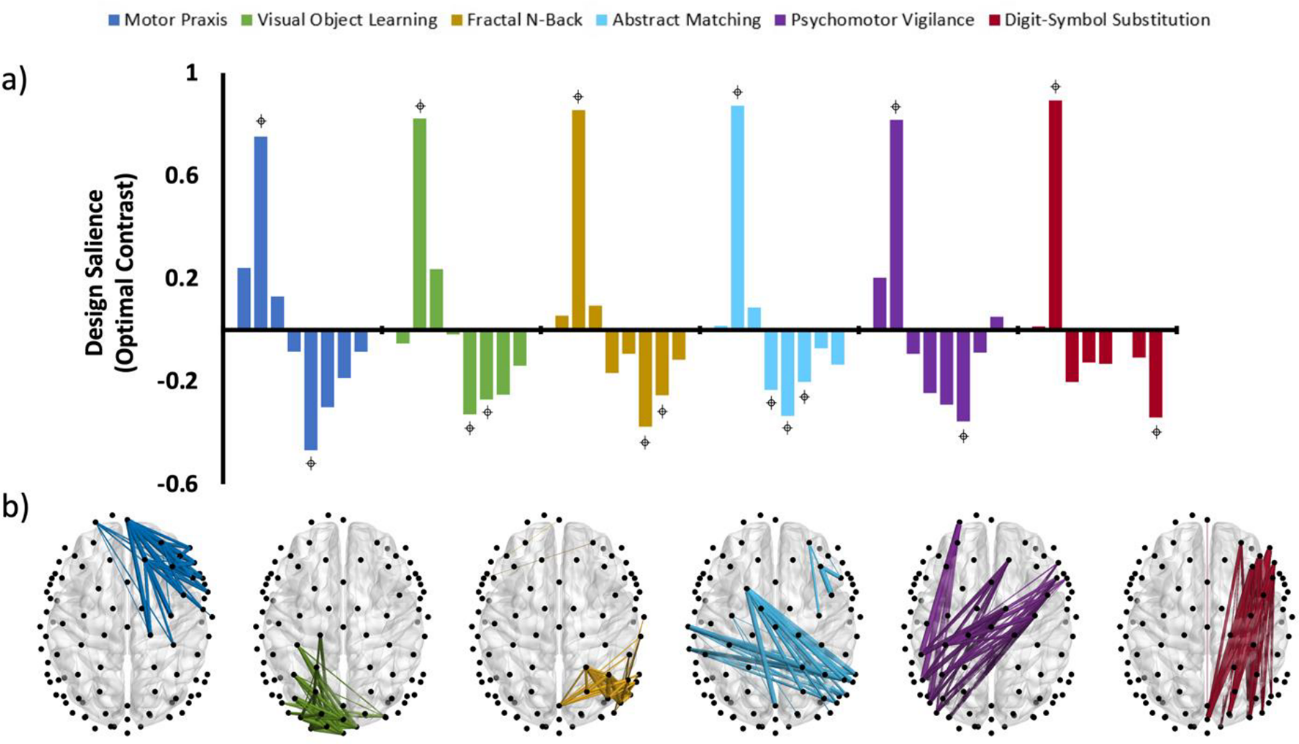


**Supplemental Figure 2.** The dominant latent variables (LV = 1 in all cases) capturing the distinct networks of maximal functional connectivity covariance across all eight cognitive testing sessions corresponding to each cognitive task in the delta bandwidth (PLS analysis, variation 3). **a)** Design saliences associated with network changes for each cognitive test across all eight sessions, where each bar corresponds to a single cognitive testing session. ⌖ = non-zero bootstrap-estimated 95% confidence interval. **b)** The top 1% of connections corresponding to each cognitive test that reliably express the contrast in the presented orientation, determined by thresholding the bootstrap ratios.


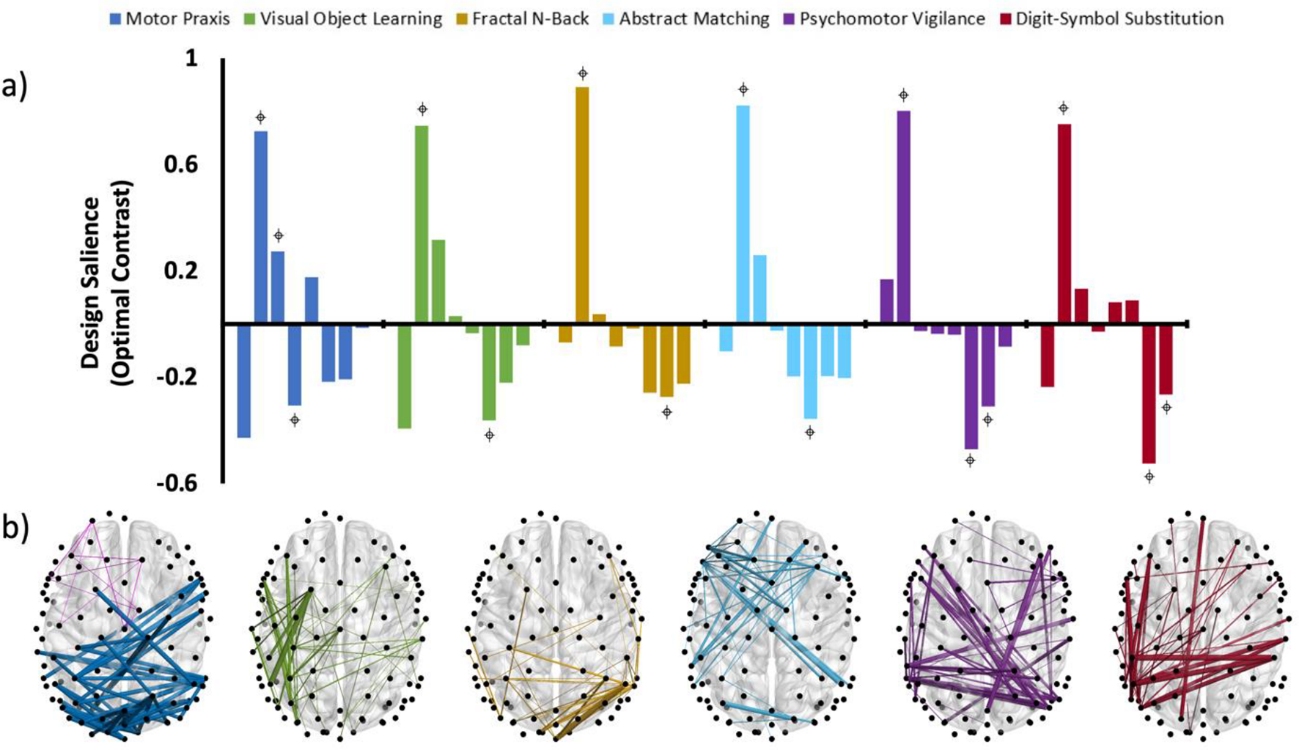


**Supplemental Figure 3.** The dominant latent variables (LV = 1 in all cases) capturing the distinct networks of maximal functional connectivity covariance across all eight cognitive testing sessions corresponding to each cognitive task in the beta bandwidth (PLS analysis, variation 3). **a)** Design saliences associated with network changes for each cognitive test across all eight sessions, where each bar corresponds to a single cognitive testing session. ⌖ = non-zero bootstrap-estimated 95% confidence interval. **b)** The top 1% of connections corresponding to each cognitive test that reliably express the contrast in the presented orientation, determined by thresholding the bootstrap ratios.


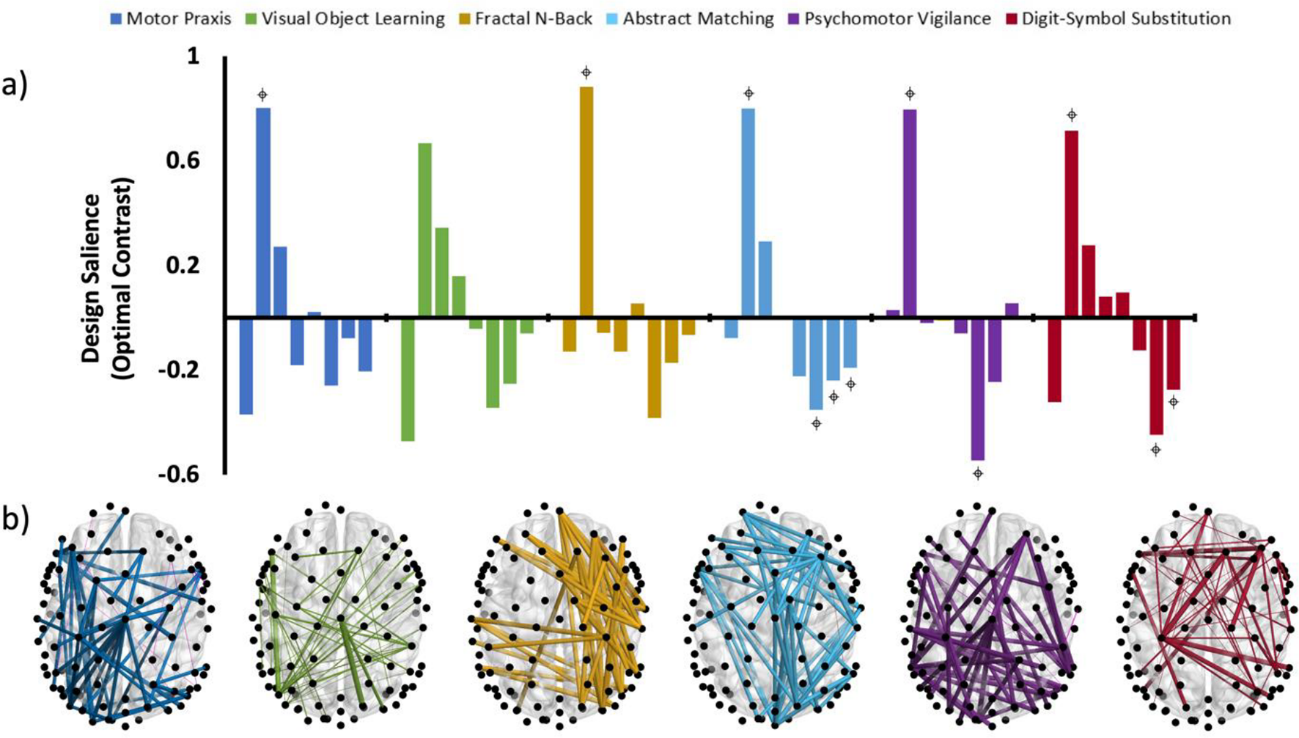


**Supplemental Figure 4.** The dominant latent variables (LV = 1 in all cases) capturing the distinct networks of maximal functional connectivity covariance across all eight cognitive testing sessions corresponding to each cognitive task in the gamma bandwidth (PLS analysis, variation 3). **a)** Design saliences associated with network changes for each cognitive test across all eight sessions, where each bar corresponds to a single cognitive testing session. ⌖ = non-zero bootstrap-estimated 95% confidence interval. **b)** The top 1% of connections corresponding to each cognitive test that reliably express the contrast in the presented orientation, determined by thresholding the bootstrap ratios.
