# Supplementary figures and images for: Distinct and Dissociable EEG Networks Are Associated With Recovery of Cognitive Function Following Anesthesia-Induced Unconsciousness
Source: Front Hum Neurosci. 2021 Sep 14;15:706693. doi: 10.3389/fnhum.2021.706693 (PMC8477048; doi:10.3389/fnhum.2021.706693)

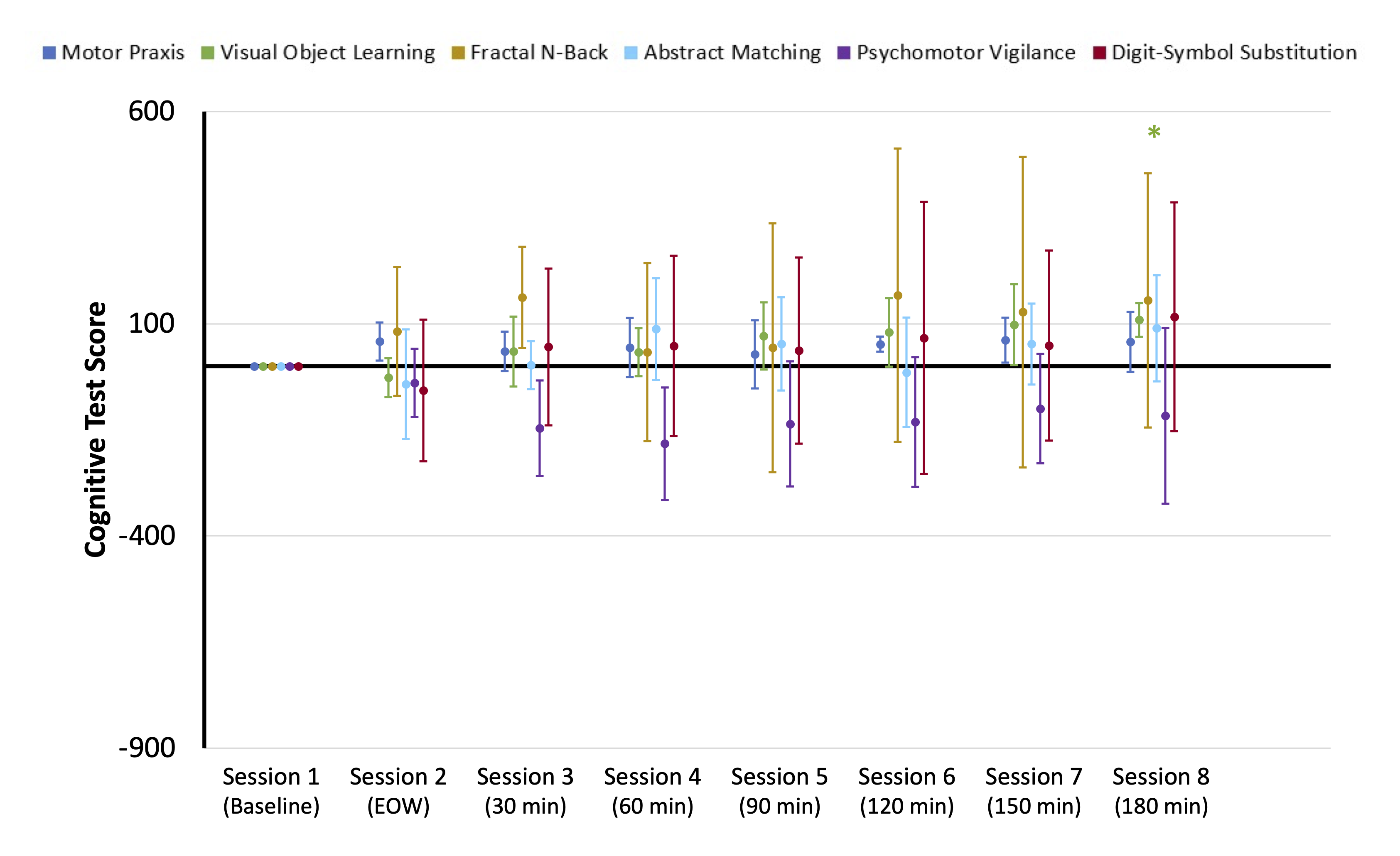

Supplement: Supplementary file 2 [file Image_1.tiff]

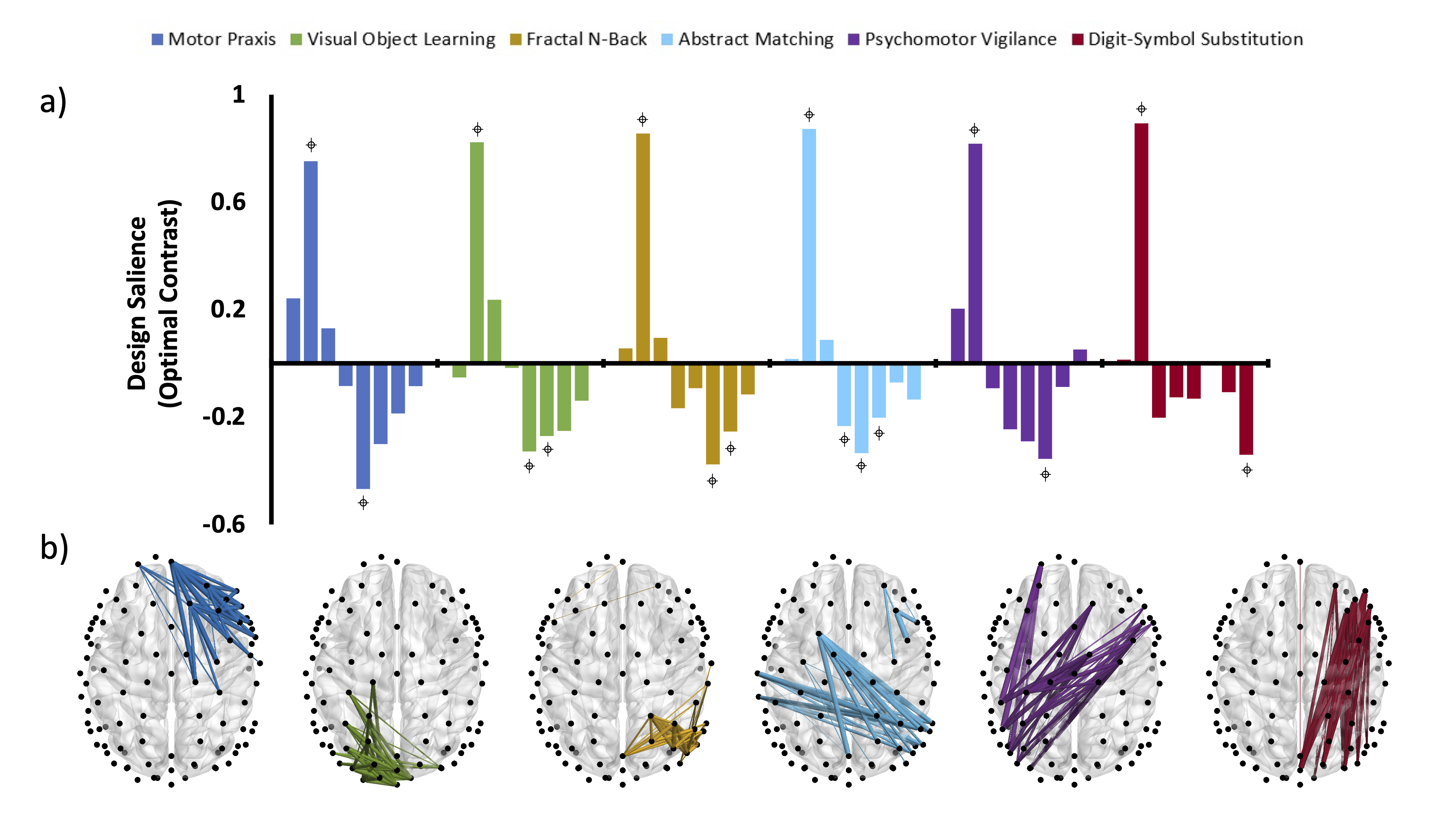

Supplement: Supplementary file 3 [file Image_2.tiff]

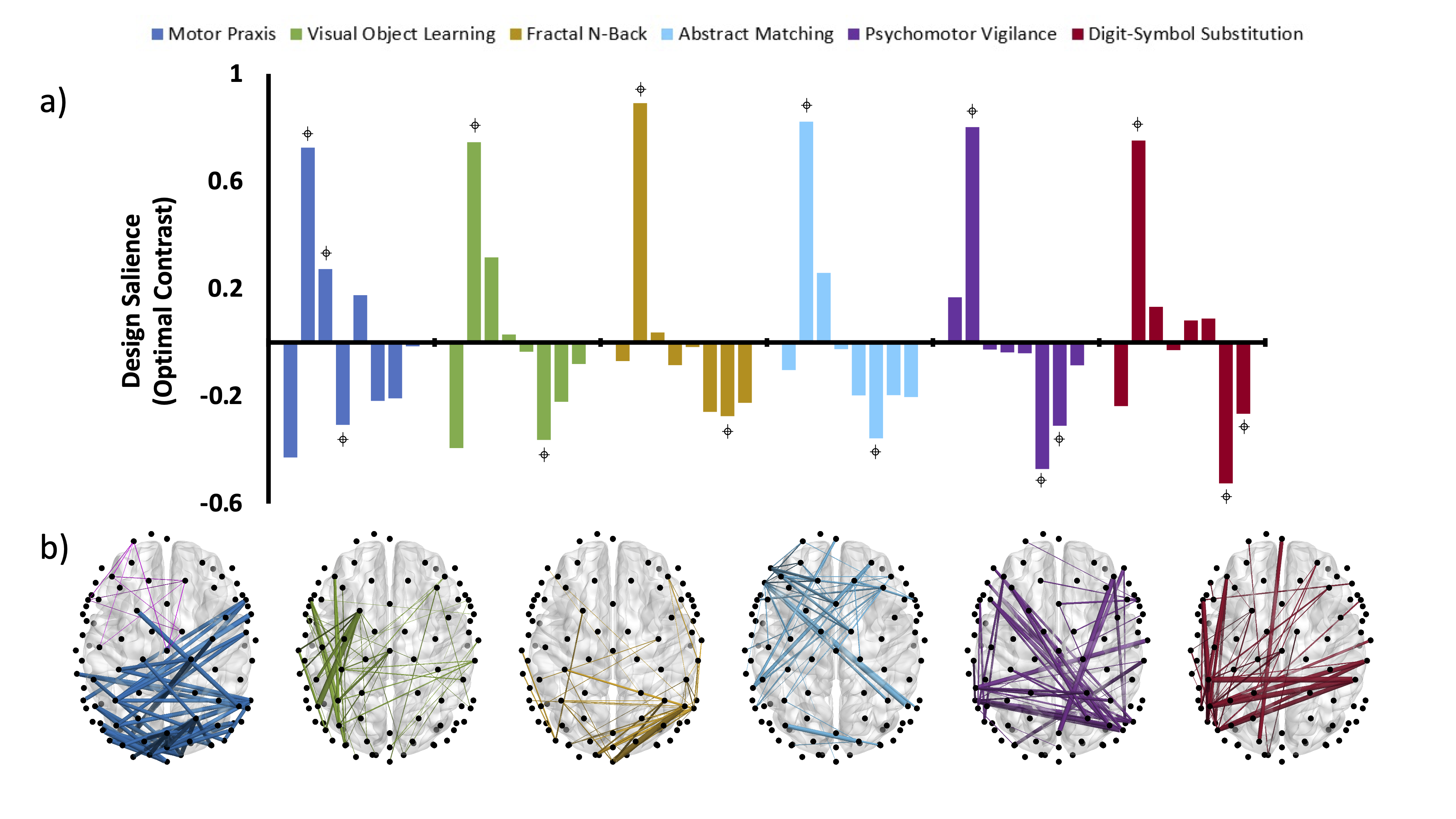

Supplement: Supplementary file 4 [file Image_3.tiff]

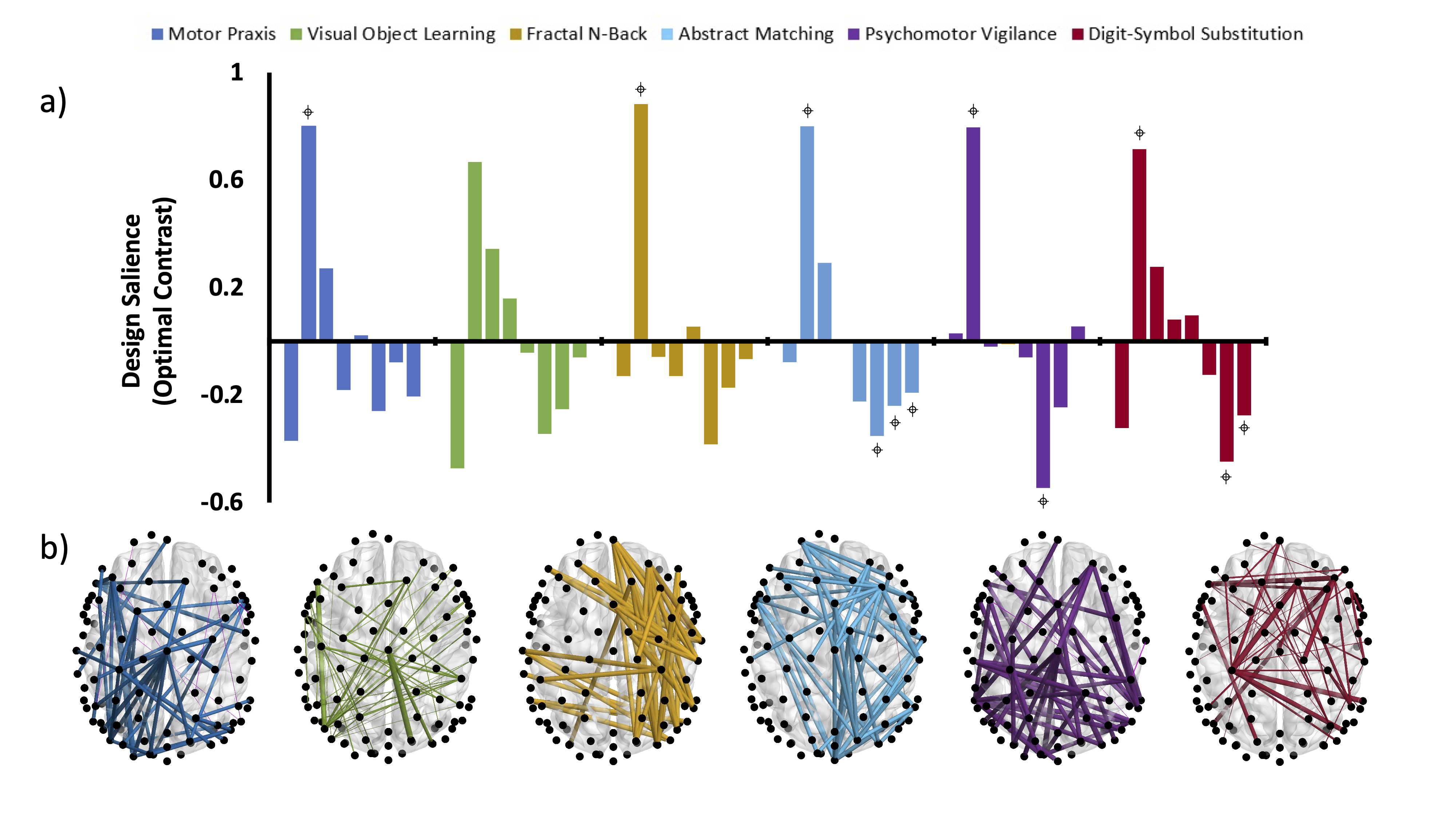

Supplement: Supplementary file 5 [file Image_4.tiff]
